# OTUB1 Recruits Tumor Infiltrating Lymphocytes and Is a Prognostic Marker in Digestive Cancers

**DOI:** 10.3389/fmolb.2020.00212

**Published:** 2020-11-06

**Authors:** Wenhao Zhang, Wenlong Qiu

**Affiliations:** ^1^Savaid Medical School, University of Chinese Academy of Sciences, Beijing, China; ^2^Qilu Hospital, Shandong University, Jinan, China

**Keywords:** digestive cancers, OTUB1, bioinformatics, prognosis, immunotherapy

## Abstract

**Background:**

The deubiquitinating enzyme (DUB) OTUB1 can regulate the process of ubiquitination, but the influence of OTUB1 on immunity, apoptosis, autophagy, and the prognosis of digestive cancers requires further exploration.

**Methods:**

OTUB1 expression was analyzed with the Oncomine and TIMER database. Kaplan-Meier plotter was used to calculate the association between OTUB1 and clinical prognosis. The regulation of OTUB1 on cancer immunocyte infiltration was determined by the TIMER database. The interaction between OTUB1 and immune genes, gene expression profiling (GEP), key genes of apoptosis and autophagy were analyzed via GEPIA. Protein-protein interaction (PPI), gene expression profiling (GEP), and functional pathway enrichment were also performed with the STRING and Pathway Common databases, respectively.

**Results:**

High OTUB1 expression was found in CHOL, LIHC, READ, ESCA, and COAD, which was significantly associated with the poorer OS of LIHC (HR = 2.07, 95% CI = 1.30–3.30, *P* = 0.002), with modifications by sex, stage, grade, and mutant burden. OTUB1 can promote the recruitment of B cells, CD8 + T cells, macrophages in ESCA, B cells, and neutrophils in LIHC. We determined a significant interaction between OTUB1 and USP8, RNF128, LRIG1, UBB, UBC, STAM2, RNF41, EGFR, RPS27A, and HGS by PPI. This functional pathway indicates the regulatory role of OTUB1on immune, apoptosis, and autophagy through its interaction with TP53 and ATG.

**Conclusions:**

OTUB1 performed as a molecular indicator of poor prognosis in digestive cancers, regulated the infiltration of tumor immunocytes, and exerted a significant influence on apoptosis and autophagy. OTUB1 is a potential antitumor target for digestive tumors.

## Introduction

Digestive cancers account for the largest number of cancer-related deaths worldwide with increasing incidence and mortality (Ferlay et al., 2015; Ahmad et al., 2017; Bray et al., 2018; Vainio, 2020). In China, digestive cancers are the highest occurring type of cancer, among which, liver, stomach, and esophageal cancer also account for a large majority of incidences. The main treatment for advanced digestive cancers is surgery combined with chemoradiotherapy, but this can have unsatisfactory outcomes, especially among patients with advanced-stage cancer, recurrence, and malnutrition, who more vulnerable and have a worse quality of life due to digestive tract dysfunction (Hartgrink et al., 2009). Thus, it is important to develop better therapeutic modalities and mechanisms to improve the prognosis of digestive cancers.

Ubiquitination is crucial in the regulation of tumor development, innate and adaptive immune responses (Hu and Sun, 2016; Fujita et al., 2019). The biological implications of ubiquitination are highlighted in homeostasis, inflammation, auto-immune and anti-tumor immunity. Apoptosis and necroptosis are two important processes in the regulation of tissue homeostasis, and their dysfunction may result in cancers and inflammatory diseases. Cell fate is also determined by immune regulation. Innate and adaptive immune receptors can mediate apoptosis and necroptosis, and subsequently, induce cell death (Peltzer and Walczak, 2019). Recent studies have demonstrated the regulatory function of ubiquitination in cell signaling pathways, inflammation, and cell death pathways (Seo et al., 2019), and further exploration of the role of the ubiquitin system in immune regulation would be beneficial to in anti-tumor immunotherapy (Aki et al., 2019). Ubiquitination also involves the control of autophagy in multiple responses. Various ubiquitin chains can promote the autophagy-dependent degradation of protein aggregates and modify the autophagy components, which is essential to regulate autophagy flux in non-selective or selective pathways (Grumati and Dikic, 2018). The concerted actions of the compounds E3 ubiquitin ligases, deubiquitinating enzymes (DUBs), and proteasome can regulate the degree of ubiquitination for specific proteins (Deshaies and Joazeiro, 2009; Komander et al., 2009). Ovarian tumor protease domain-containing DUBs (OTUDs) are cysteine-dependent proteases associated with poor cellular functions, and the role of OTUDs enzymes in carcinogenesis is emerging gradually. Of which, ovarian tumor domain-containing ubiquitin aldehyde binding protein 1 (OTUB1) is an atypical DUB in the regulation of specific ubiquitin-conjugating enzymes (E2s). It can directly cleave the ubiquitin chains from target proteins, and subsequently, bind and inhibit the ubiquitination of E2s independently of their enzymatic activity (Wang et al., 2009; Nakada et al., 2010; Juang et al., 2012; Wiener et al., 2012, 2013; Gubser et al., 2013). The effect of OTUB1 on tumors is complex. As a tumor suppressor, OTUB1 can inhibit cell growth and induce P53-dependent apoptosis through the regulation of DNA damage response (Nakada et al., 2010; Sun et al., 2012; Kato et al., 2014). However, OTUB1 also performed crucially in TGF-β-mediated gene transcription and cellular migration, subsequently promote tumor migration through (Herhaus et al., 2013). There are still some debates on the biological role played by OTUB1 in tumorigenesis.

At present, research on digestive cancers has largely focused on the role of OTUB1 in regulating the ubiquitination process, with few studies dedicated to clarifying the function of OTUB1 in autophagy, apoptosis, and the immune microenvironment. In this study, we systematically analyzed the expression, prognostic value, and function of OTUB1 with bioinformatics databases, to enrich understanding of OTUB1.

## Materials and Methods

### Oncomine Database Analysis

We identified the different expression levels of the OTUB1 gene in digestive cancers with the Oncomine database^1^ (Rhodes et al., 2007), and defined the threshold according to the following values: *P*-value of 0.001, fold change of 1.5, and gene ranking of all.

### TIMER Database Analysis

TIMER is an online database for the systematic analysis of immune infiltrates in diverse cancer types^2^ (Li et al., 2017). The expression of OTUB1 in different cancers and the correlation with immune infiltrates was analyzed via gene modules, including B cells, CD4 + T cells, CD8 + T cells, neutrophils, macrophages, and dendritic cells (Aran et al., 2015). A Copy Number Variations (CNV) module was applied to analyze the correlation between gene alterations and tumor infiltration levels in specific tumors. The CNV was defined by GISTIC 2.0, including deep deletion, arm-level deletion, diploid/normal, arm-level gain, and high amplification. We compared the infiltration level of each CNV category with the normal via the Wilcoxon rank-sum test. The associations between CNV types of OTUB1 and lymphocytes infiltration levels were also analyzed.

### Kaplan-Meier Plotter Database Analysis

Kaplan-Meier Plotter is an online tool that can assess the effect of genes on survival in at least 10,000 cancer samples. We analyzed the correlation between OTUB1 expression and the survival of digestive cancers in pan-cancers with the Kaplan-Meier Plotter^3^ (Lánczky et al., 2016). We also analyzed the relationship of OTUB1 expression with overall survival (OS) and relapse-free survival (RFS) in liver hepatocellular carcinoma (LIHC), esophageal adenocarcinoma (ESAD), esophageal carcinoma (ESCA), stomach adenocarcinoma (STAD), pancreatic adenocarcinoma (PAAD), and rectum adenocarcinoma (READ). Hazard Ratios (HRs) with 95% confidence intervals (CI) and log-rank *P* value were calculated.

### Gene Correlation Analysis in GEPIA

Gene Expression Profiling Interactive Analysis (GEPIA)^4^ (Tang et al., 2017) is an online database of 9,736 tumors, with 8,587 normal samples. Using data from TCGA and the GTEx projects, along with the analysis of RNA sequencing expression, we analyzed the association between OTUB1 expression and the marker genes of tumor-infiltrating immunocytes, mainly including T cell, TAM, macrophage, NK cells, DC cell, T-help cells, and neutrophils (Sousa and Määttä, 2016; Danaher et al., 2017; Siemers et al., 2017). We performed an analysis of gene expression correlation for given sets with TCGA expression data. The correlation of OTUB1 and other genes was compared between tumor and normal tissue datasets. We set OTUB1 as the *X*-axis and other genes of interest as the *Y*-axis. The correlation coefficient was determined by the Spearman method.

### Protein-Protein Interaction (PPI), Gene Regulation, and Functional Pathway Enrichment Analysis

The search Tool for the Retrieval of Interacting Genes (STRING) database was designed to evaluate the protein-protein interaction (PPI) information, and visualize the functional interactions between the above proteins (Szklarczyk et al., 2015). The Pathway Commons database^5^ is an integrated resource of public biological pathways with various biochemical reactions. The database contains about 500 detailed human biochemical processes and millions of interactions. To enhance the usability of this large resource for end-users, researchers develop and maintain interactive web applications and training materials to enable pathway exploration and advanced analysis (Rodchenkov et al., 2020). We performed functional enrichment analysis of OTUB1 with the Pathway Common database. The main content of the analysis includes gene interaction network construction and functional pathway enrichment analysis.

### Statistical Analysis

Kaplan-Meier plots were used to generate survival curves and displayed the results with HR and *P* or Cox *P*-values from a log-rank test. The correlation of gene expression was evaluated with Spearman’s correlation analysis. *P*-values < 0.05 were considered statistically significant.

## Results

### The Different Expression Level of OTUB1 mRNA in Digestive Cancers

**FIGURE 1:**
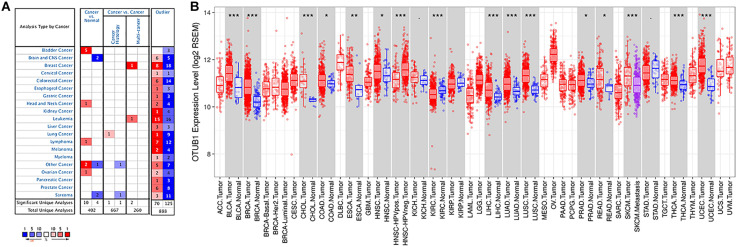
The expression level of OTUB1 in various cancers. **(A)** The comparison of the expression of OTUB1 between cancer tissues and normal tissues in ONCOMINE. The number of each dataset was also shown. **(B)** The expression level of OTUB1 in different cancers in the TCGA database, analyzed with TIMER. *P < 0.05, **P < 0.01, ***P < 0.001.

The expression levels of OTUB1 mRNA in digestive tumors and normal tissues were compared with the Oncomine database. No significant expression differences were observed in the above digestive tract tumors (Figure 1A), except for bladder cancer. Otherwise, the high expression of OTUB1 was observed in head and neck, ovarian and lymphoma tumors compared to the normal tissues in some data sets. The expression of OTUB1 between the tumor and adjacent normal tissues was further evaluated in digestive cancers with the TIMER database (Figure 1B). The expression of OTUB1 was significantly higher in the tumor tissue of CHOL, LIHC, READ, ESCA, and COAD compared with their adjacent normal tissues.

### High OTUB1 Expression Is Associated With Poor Prognosis

**FIGURE 2:**
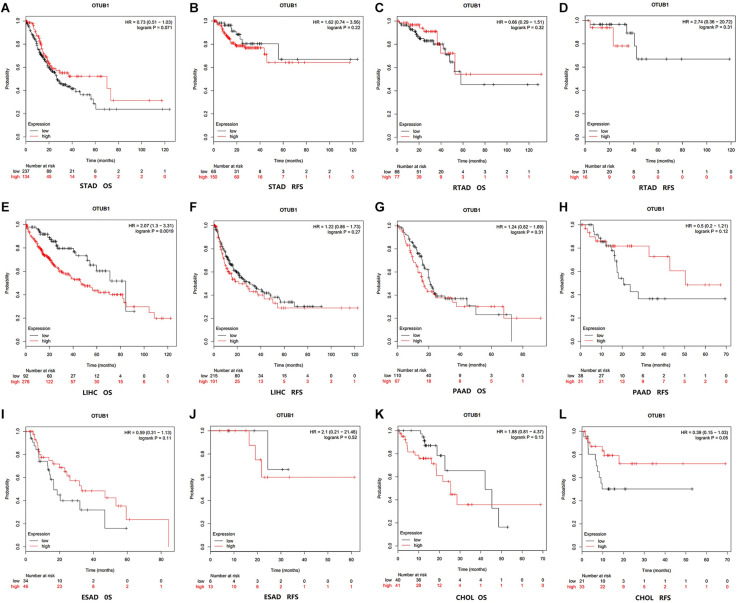
Kaplan-Meier survival curves visualized the expression level of OTUB1 in different cancers with Kaplan-Meier Plotter database. **(A)** OS (*n* = 371) in STAD cohort. **(B)** RFS (*n* = 215) in STAD cohort. **(C)** OS (*n* = 165) in RTAD cohort. **(D)** RFS (*n* = 49) in RTAD cohort. **(E)** OS (*n* = 370) in LIHC cohort. **(F)** RFS (*n* = 316) in LIHC cohort. **(G)** OS (*n* = 177) in PAAD cohort. **(H)** RFS (*n* = 69) in PAAD cohort. **(I)** OS (*n* = 80) in ESAD cohort. **(J)** RFS (*n* = 19) in ESAD cohort. **(K)** OS (*n* = 81) in CHOL cohort. **(L)** RFS (*n* = 54) in CHOL cohort.

**FIGURE 3:**
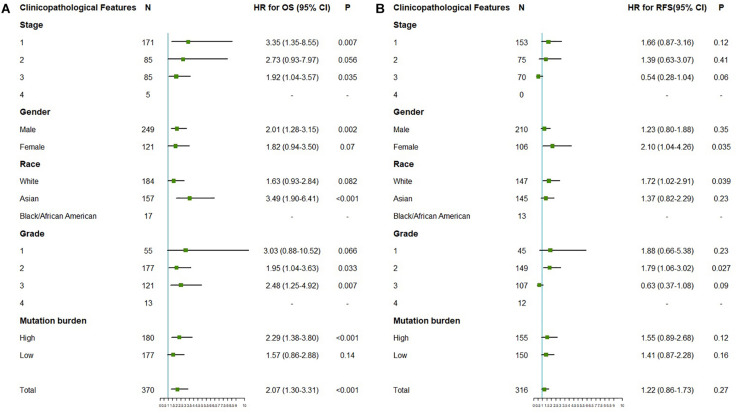
The correlation between the expression of OTUB1 mRNA and OS (*n* = 370, **A**), PFS (*n* = 316, **B**) in LIHC. The analysis was stratified by different clinicopathological features. Green squares represent HR. LIHC, liver hepatocellular carcinomas; HR, hazard ratio; OS, overall survival; RFS, relapse-free survival.

The correlation between OTUB1 expression and the prognosis (overall survival and relapse-free survival) of digestive cancer patients was investigated with the Kaplan-Meier plotter database (Figures 2A–L). High OTUB1 expression significantly interferes with the overall survival (OS) of LIHC (Figure 2E) and the relapse-free survival (RFS) of ESCA (Figure 2L). Our study showed that high OTUB1 expression was associated with poorer OS (HR = 2.07, 95% CI = 1.30 to 3.30, *P* = 0.002) in LIHC cohorts including 370 samples in the pan-cancer dataset (Figure 3). The high expression of OTUB1 was related to a poor OS in Asian (HR = 3.49, 95%CI = 1.90–6.41, *P* < 0.001) and male (HR = 2.01, 95%CI = 1.28–3.15, *P* = 0.0019) patients, and patients with stage 1 (HR = 3.35, 95%CI = 1.35–8.55, *P* = 0.0073), stage 3 (HR = 1.92, 95%CI = 1.04–3.57, *P* = 0.035), grade 2 (HR = 1.95, 95%CI = 1.28–3.15, *P* = 0.0019), grade 3 (HR = 2.48, 95%CI = 1.25–4.92, *P* = 0.0073), and high mutation burden (HR = 2.29, 95%CI = 1.38–3.80, *P* < 0.001; Figure 3A). The total RFS was not affected by OTUB1 expression, however, in the White (HR = 1.72, 95%CI = 1.02–2.91, *P* = 0.039) and female (HR = 2.10, 95%CI = 1.04–4.26, *P* = 0.035) patients, and patient with grade 2 (HR = 1.79, 95%CI = 1.06–3.02, *P* = 0.027), the high expression of OTUB1 means a poor RFS (Figure 3B).

### OTUB1 Expression Regulate the Level of Immune Infiltration in Digestive Cancers

**FIGURE 4:**
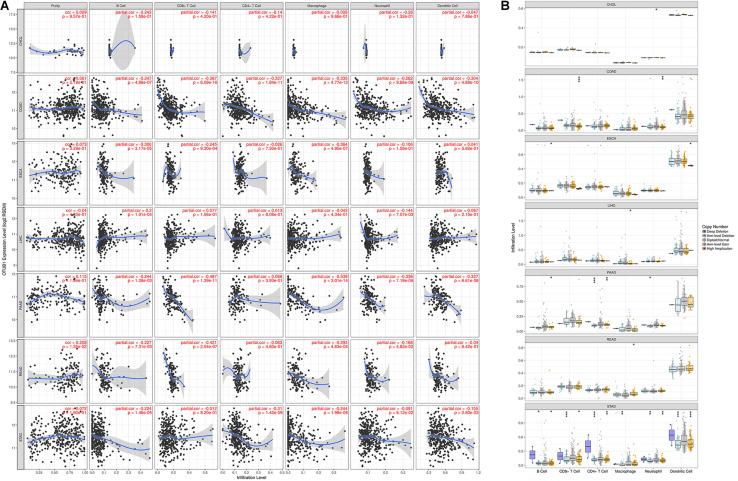
OTUB1 expression is correlated with the infiltration level of immune cells in digestive cancers. **(A)** OTUB1 expression has no relation with tumor purity and significant positive correlation with infiltrating levels of B cell, macrophage, neutrophil, CD8 +, and CD4 + T cell, dendritic cell. **(B)** The CNV of OTUB1 significantly changes the lymphocyte infiltration level.

The results show that the expression of OTUB1 in READ has significant correlations with tumor purity. The OTUB1 expression in READ also correlates with the infiltration levels of CD8 + T, CD4 + T, B cell, macrophage, neutrophil, and dendritic cells. OTUB1 expression in ESCA has significant correlations with the infiltrating levels of B cells, CD8 + T cells, and macrophages, and OTUB1 expression in LIHC has significant correlations with the infiltrating levels of B cells and neutrophils. The correlations between OTUB1 expression and tumor-infiltrating lymphocytes in PAAD, READ, and STAD are shown in Figure 4A. To further explore the association between the expression level of OTUB1 and tumor-infiltrating lymphocytes in different digestive cancers, we analyzed the effect of the copy number variation (CNV) of OTUB1 on the infiltration level of tumor-infiltrating lymphocytes (Figure 4B). Among the CNV types, the arm-level gain is the most common form of mutation of the OTUB1 gene. The arm-level gain mutation of the OTUB1 gene in COAD, ESCA, and PAAD can modify the infiltration level of B cells. The arm-level gain can also influence the infiltration level of specific lymphocytes in several cancers. Other forms of mutations can also alter the infiltration level of lymphocytes. High amplification variation of the OTUB1 gene can elevate the infiltration level of macrophages in LIHC and STAD. We speculated that the infiltration level of lymphocytes could be modified by CNV of the OTUB1 gene and further proved the role of OTUB1 in the modification of TILs.

### OTUB1 Expression Was Correlated With Gene Marker Sets of Tumor-Infiltrating Lymphocytes

The relationship between OTUB1 and immune infiltrating cells was investigated through analysis of the correlations between OTUB1 and the gene marker sets of immune cells in the tumor and normal tissues with GEPIA databases. This analysis was focussed on LIHC and ESCA, which are the major cancers of digestive cancers. The immune cells of interest included in our analysis were NK cells, DC cells, TAM, monocytes, neutrophils, B cells, M1, M2 macrophages, and different functional T cells (Table 1). We found that the expression level of OTUB1 can significantly impact the expression of most of the immune marker sets of various immune cells in both tumor and cancer tissues. To further analyze the regulatory effect of OTUB1 expression on the immune marker sets of immune cells in tumor tissues, we compared the differences of this correlation between tumor and normal tissues. The marker genes of immune cells in ESCA tumor tissues are more susceptible to OTUB1 than those of immune cells in normal tissues. The regulatory ability of OTUB1 to immune cell genes in tumor tissues and normal tissues is significantly different. However, in LIHC tumor tissues, OTUB1 was not significantly related to immune cell marker genes compared with normal tissues. Specifically, we found that the CX3CR1 of effector T cells, CXCL13 of exhausted T cells, CXCL13, BHLHE40, CD4 of Th1-like cells, KIR2DL3, KIR2DL4, KIR3DL2, KIR3DL3 of NK cells, and the HLA-DQB1 of DC cells are significantly correlated with OTUB1 expression in LIHC. These results revealed that OTUB1 can specifically influence the level and types of immune infiltrating cells in LIHC and ESCA, and subsequently modify the tumor microenvironment.

**TABLE 1:** Correlations between OTUB1 and gene markers of immune cells in GEPIA.

**Cell types**	**Gene markers**	**LIHC**				**ESCA**			
		**Tumor**		**Normal**		**Tumor**		**Normal**	
		**P**	**R**	**P**	**R**	**P**	**R**	**P**	**R**
Naïve T-cell	CCR7	0.0024	0.16	1.5e-07	0.66	8.1e-05	–0.29	0.053	0.55
	LEF1	7.7e-11	0.33	0.00037	0.48	0.94	0.0054	0.0069	0.73
	TCF7	8e-05	0.2	5.6e-05	0.54	0.52	0.048	0.078	0.51
	SELL	1.7e-06	0.25	4.6e-05	0.54	5.6e-05	–0.29	0.15	0.43
Effector T-cell	CX3CR1	1.6e-10	0.33	0.11	0.23	0.00097	–0.24	0.012	0.69
	FGFBP2	0.36	–0.048	0.0042	0.4	0.063	0.14	0.064	0.53
	FCGR3A	3.6e-05	0.21	0.003	0.41	0.46	–0.055	0.57	0.17
	CD8A	7e-04	0.18	1.2e-05	0.58	0.0021	–0.23	0.89	0.041
	CD8B	0.00068	0.18	2.2e-06	0.61	0.0012	–0.24	0.77	–0.091
Effector memory T-cell	PDCD1	5.2e-09	0.3	0.00038	0.48	0.051	–0.14	0.15	0.43
	DUSP4	5.3e-09	0.3	0.00013	0.52	0.0013	–0.24	0.32	–0.3
	GZMK	0.059	0.098	2.2e-06	0.61	1.4e-06	–0.35	0.62	0.15
	GZMA	0.05	0.1	6.1e-05	0.54	0.064	–0.14	0.35	0.28
	IFNG	0.00018	0.19	0.034	0.3	0.07	–0.13	0.86	–0.053
Resident memory T-cell	CD69	0.0013	0.17	0.00083	0.46	0.0019	–0.23	0.52	0.2
	ITGAE	5.8e-16	0.4	0.0044	0.4	0.00024	0.27	0.0033	0.77
	CXCR6	0.0064	0.14	3.3e-06	0.6	4e-05	–0.3	0.71	0.11
	MYADM	4.1e-16	0.41	0.014	0.34	0.044	–0.15	0.71	0.12
Central memory T-cell	CCR7	0.0024	0.16	1.5e-07	0.66	8.1e-05	–0.29	0.053	0.55
	SELL	1.7e-06	0.25	4.6e-05	0.54	5.6e-05	–0.29	0.15	0.43
	IL7R	0.03	0.11	3.3e-05	0.55	0.43	–0.058	0.5	0.21
Exhausted T-cell	HAVCR2	1.4e-08	0.29	0.00044	0.48	0.018	–0.17	0.11	0.47
	GZMB	0.08	0.091	9.2e-06	0.58	0.46	–0.055	0.18	0.4
	TIGIT	5.7e-06	0.23	0.00036	0.49	0.00067	–0.25	0.12	0.45
	LAG3	0.069	0.095	0.0022	0.42	0.46	–0.056	0.038	0.59
	PDCD1	5.2e-09	0.3	0.00038	0.48	0.051	–0.14	0.15	0.43
	CXCL13	0.032	0.11	0.2	0.18	0.013	–0.18	0.7	0.12
	LAYN	1.1e-10	0.33	0.00044	0.48	0.14	0.11	0.16	0.42
Resting Treg	FOXP3	0.42	0.042	0.019	0.33	0.0033	–0.22	0.054	0.54
	IL2RA	1.1e-07	0.27	0.038	0.29	0.072	–0.13	0.061	0.54
Effector Treg	CTLA4	9.1e-06	0.23	0.0022	0.42	0.014	–0.18	0.12	0.45
	CCR8	1.6e-07	0.27	0.035	0.3	0.00095	–0.24	0.072	0.51
	TNFRSF9	8.8e-10	0.31	0.0073	0.38	0.19	–0.099	0.11	0.46
Th1-like	CXCL13	0.032	0.11	0.2	0.18	0.013	–0.18	0.7	0.12
	HAVCR2	1.4e-08	0.29	0.00044	0.48	0.018	–0.17	0.11	0.47
	IFNG	0.00018	0.19	0.034	0.3	0.07	–0.13	0.86	–0.053
	CXCR3	1.1e-05	0.23	6.7e-05	0.53	3.4e-05	–0.3	0.28	0.32
	BHLHE40	0.005	0.15	0.58	–0.079	0.88	0.011	0.004	0.76
	CD4	0.00023	0.19	0.19	0.19	3e-04	–0.27	0.45	0.23
	TNF	8e-09	0.29	0.0029	0.41	0.085	0.13	0.0036	0.76
	Tbx21	0.057	0.099	3.7e-07	0.65	0.00019	–0.27	0.58	0.17
	STAT4	3.5e-05	0.21	0.0018	0.43	0.00038	–0.26	0.45	0.23
	STAT1	2.3e-17	0.42	1.4e-05	0.57	0.25	0.086	0.004	0.76
Natural killer cell	KIR2DL1	0.67	0.022	0.16	0.2	0.13	–0.11	0.32	0.3
	KIR2DL3	0.0032	0.15	0.072	0.26	0.093	–0.13	0.4	0.25
	KIR2DL4	0.0012	0.17	0.079	0.25	0.7	–0.029	0.92	–0.03
	KIR3DL1	0.61	–0.026	0.0027	0.42	0.0085	–0.19	0.76	0.096
	KIR3DL2	2e-04	0.19	0.76	–0.044	0.06	–0.14	0.089	–0.49
	KIR3DL3	0.024	0.12	0.44	0.11	0.75	–0.023	0.45	0.23
	KIR2DS4	0.2	0.066	0.87	0.024	0.32	–0.074	0.54	–0.19
Th17	STAT3	1.2e-06	0.25	0.73	0.049	0.4	0.063	0.0026	0.78
	IL17A	0.99	0.00059	0.35	0.13	0.41	–0.061	0.13	0.44
Th2	GATA3	6e-05	0.21	0.0079	0.37	0.039	–0.15	0.054	0.54
	STAT6	4.5e-05	0.21	3.2e-07	0.65	0.91	0.0089	0.0033	0.77
	STAT5A	4.4e-11	0.33	1.2e-10	0.76	0.16	–0.11	0.022	0.64
	IL13	0.11	0.082	0.041	0.29	0.039	–0.15	0.52	0.2
Dendritic cell	HLA-DPB1	7.6e-09	0.29	1.2e-08	0.7	0.00035	–0.26	0.093	0.49
	HLA-DQB1	0.00089	0.17	0.58	0.079	0.018	–0.17	0.089	0.49
	HLA-DRA	6.2e-08	0.28	4e-07	0.65	5e-04	–0.26	0.034	0.6
	HLA-DPA1	7.2e-07	0.25	2.4e-06	0.61	0.0012	–0.24	0.061	0.54
	CD1C	2.3e-07	0.27	0.0054	0.39	0.014	–0.18	0.061	0.54
	NRP1	7.5e-09	0.3	0.076	0.25	0.088	–0.13	0.46	0.22
	ITGAX	4.1e-09	0.3	0.0022	0.42	0.0015	–0.23	0.1	0.48
TAM	CCL2	2.8e-05	0.22	0.011	0.36	0.18	–0.099	0.14	0.43
	CD68	6.4e-07	0.26	3.8e-08	0.69	0.96	0.0036	0.0036	0.76
	IL10	0.00026	0.19	0.0016	0.44	0.36	–0.068	0.16	0.41
Monocyte	CD86	1.8e-09	0.31	1.7e-06	0.62	0.32	–0.075	0.14	0.43
	CD115	7.5e-10	0.31	3.7e-05	0.55	0.072	–0.13	0.25	–0.34
Neutrophil	CEACAM8	0.11	0.084	0.053	0.28	0.14	–0.11	0.32	0.3
	ITGAM	1.6e-12	0.36	8.3e-07	0.63	0.13	–0.11	0.33	0.29
	CCR7	0.0024	0.16	1.5e-07	0.66	8.1e-05	–0.29	0.053	0.55
B cell	CD19	0.00061	0.18	0.0034	0.41	0.00023	–0.27	0.093	0.49
	CD79A	0.0029	0.15	0.00078	0.46	2.5e-05	–0.31	0.91	–0.038
M1 macrophage	NOS2	0.33	0.05	0.00068	0.46	0.0057	–0.2	0.026	0.61
	IRF5	2.4e-14	0.38	4.2e-05	0.55	0.29	0.078	0.00044	0.85
	PTGS2	4.1e-05	0.21	0.083	0.25	0.056	0.14	0.05	0.56
M2 macrophage	CD163	0.52	0.034	0.00093	0.45	0.085	–0.13	0.85	0.06
	VSIG4	0.00011	0.2	0.00022	0.5	0.55	–0.044	0.72	–0.11
	MS4A4A	0.0031	0.15	1.2e-05	0.58	0.0018	–0.23	0.46	–0.23

### Protein-Protein Interaction (PPI) and Gene Expression Profiling (GEP) Analysis

**FIGURE 5:**
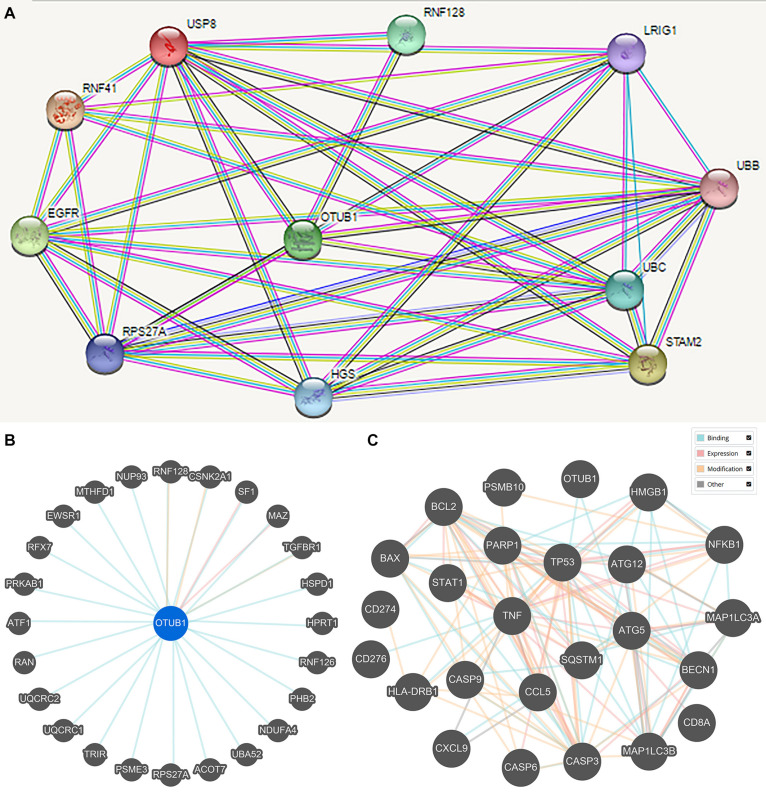
The STRING online database was applied to obtain the PPI information of the DEGs. **(A)** The prominent proteins were identified after PPI analysis with STRING. **(B)** Interactions between OTUB1 and 25 other genes. **(C)** Interaction between OTUB1 and the key genes of GEP, apoptosis, and autophagy.

Protein-protein interaction (PPI) enrichment was constructed with the STRING database. The 10 most significant correlated genes of OTUB1 were USP8, RNF128, LRIG1, UBB, UBC, STAM2, RNF41, EGFR, RPS27A, and HGS (Figure 5A). OTUB1 can regulate T-cell anergy via interaction with RNF128/GRAIL. RNF128 is an important inductor of anergic phenotype. It can induce CD4 T-cell anergy and inhibit the transcription of cytokine genes, which is associated with the blockade of interleukin production (Lin et al., 2009; Stempin et al., 2017). USP8 can form a complex with RNF128 and OTUB1 to regulate the anergy of T-cells mediated by RNF128 (Dufner et al., 2015). STAM2 and HGS are two crucial regulators in intracellular signal transduction, a process that is modified by cytokines and growth factors (Endo et al., 2000; Yamada et al., 2002; Flores-Rodriguez et al., 2015). Further analysis revealed that OTUB1 played a major biological role in regulating ubiquitination-related synthesis and degradation (Figure 5B). To further study the role of OTUB1 in the regulation of immunity, apoptosis, and autophagy, we analyzed the influence of OTUB1 expression on the marker genes of gene expression profiles (GEPs), apoptosis, and autophagy of TCGA-LIHC dataset via GEPIA (Table 2). We also calculated and visualized the interaction between them (Figure 5C). We found that OTUB is closely related to key proteins in the regulation of apoptosis and autophagy. Specifically, OTUB1 can directly bind to TP53, thereby regulating the apoptosis process of cells and promoting the expression of apoptotic genes. OTUB1 is also directly combined with ATG5 to regulate the expression of autophagy-related genes, such as BECN, SQSTM1, and MAP1LC3A. The OTUB1 gene was correlated with the expression of most of the immune-related genes listed in Table 2, which can be regulated by OTUB1 through direct and indirect channels. Therefore, the expression of OTUB1 exerted a significant regulatory effect on the expression of GEP-related genes.

**TABLE 2:** Correlations between OTUB1 and the key gene markers of GEP, apoptosis, and autophagy in GEPIA.

**Gene markers**	**Tumor**		**Normal**	
	**P**	**R**	**P**	**R**
CXCR6	0.0064	0.14	3.3e-6	0.6
TIGIT	5.7e-6	0.23	0.00036	0.49
CD27	0.00011	0.2	5.4e-5	0.54
PDCD1LG2 (PD-L2)	0.14	0.078	0.00074	0.46
CD274 (PD-l1)	0.00035	0.19	0.00039	0.48
CD8A	7e-4	0.18	1.2e-5	0.58
LAG3	0.069	0.095	0.0022	0.42
NKG7	0.55	0.032	1.5e-6	0.62
CCL5	0.012	0.13	1.5e-6	0.62
CMKLR1	0.00017	0.19	0.00037	0.48
PSMB10	0.00086	0.17	5.3e-9	0.72
CXCL9	0.0023	0.16	7.8e-5	0.53
IDO1	0.0033	0.15	0.00028	0.49
HLA-DQA1	2e-8	0.29	0.00049	0.48
HLA-DRB1	3.5e-6	0.24	3.6e-5	0.55
HLA-E	4e-5	0.21	6e-9	0.71
CD276	5.1e-33	0.57	0.052	0.28
STAT1	2.3e-17	0.42	1.4e-5	0.57
BCL2	4.8e-7	0.26	1.4e-5	0.57
BAX	4.7e-24	0.49	3.7e-8	0.69
CASP3	1.9e-17	0.42	0.00021	0.5
CASP6	2.9e-18	0.43	2e-4	0.5
CASP9	1.4e-6	0.25	7.1e-8	0.68
TNF	8e-9	0.29	0.00029	0.41
NFKB1	1.9e-14	0.38	0.0015	0.44
HMGB1	5.7e-17	0.42	1.4e-5	0.57
PARP1	3e-22	0.48	0.00073	0.46
TP53	5e-15	0.39	0.00039	0.48
DRAM	3.9e-31	0.55	6.5e-06	0.59
SQSTM1 (P62)	4.5e-7	0.26	5.4e-6	0.59
BECN1 (Beclin1)	3.6e-27	0.52	1.3e-8	0.7
MAP1LC3A (LC3A)	0.23	−0.063	0.0074	0.37
MAP1LC3B (LC3B)	6.4e-8	0.28	0.0019	0.43
ATG5	6.3e-11	0.33	2.3e-5	0.56
ATG12	1.4e-12	0.36	2.7e-5	0.56

### Functional Pathway Enrichment Analysis

**FIGURE 6:**
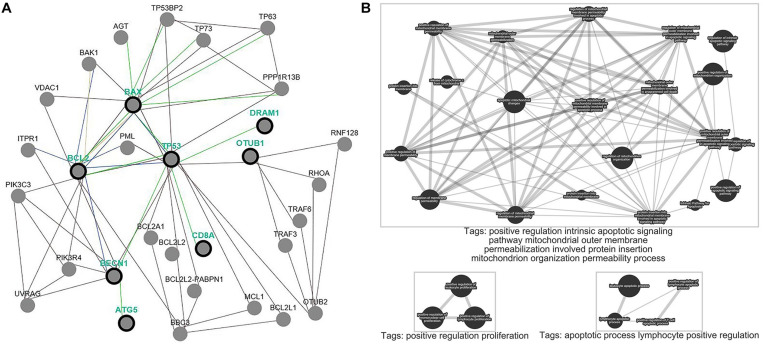
Pathway common network and enrichment analyses of OTUB1. **(A)** Pathway common network of OTUB1 and BCL-2, BAX, TP53, DRAM1, BECN, ATG5, CD8A. **(B)** Pathway enrichment analysis of OTUB1, based on the key genes of immune, apoptosis, and autophagy.

We performed analyses based on the gene-level to clarify the function of OTUB1 on the pathway-level. We selected OTUB1 and apoptotic genes BCL-2, BAX, autophagy genes TP53, DRAM, BECN, ATG5, and immune gene CD8A to clarify the effect of OTUB1 on the regulation of the above cytology functions (Figure 6A). The results revealed that OTUB1 directly regulated the TP53 and CD8A genes. A functional enrichment analysis showed that OTUB1 positively regulated the intrinsic apoptotic signaling pathway, and increased the permeability of the mitochondrial outer membrane, and consequently promoted the protein insert into the mitochondrial organization. What’s more, OTUB1 performed a dual effect on the fate of leukocyte, lymphocytes, and mononuclear cells (Figure 6B). OTUB1 might also regulate the autophagy through the P53-DRAM pathway, which is a damage-related autophagy pathway. Above all, OTUB1 is an important regulator in the immune, apoptosis, and autophagy processes.

## Discussion

OTUB1 is a de-ubiquitinating enzyme (DUB) that belongs to the OUT-protein superfamily. It can remove the ubiquitin from the proteins and control protein transformation through the regulation of protein degradation (Soares et al., 2004; Wiener et al., 2012) and is a valuable prognostic biomarker for several cancers (Zhou et al., 2014; Wang et al., 2016; Weng et al., 2016). This study revealed that OTUB1 expression was associated with a poorer OS of LIHC and the RFS of ESCA. Furthermore, our analyses revealed that OTUB1 could modify the level and types of immune infiltrating lymphocytes. Furthermore, OTUB1 regulated the process of autophagy and immunity in cells, which is a topic of particular interest in molecular biology research. Although the prognosis of gastrointestinal tumors has been significantly improved by comprehensive treatments, the prognosis of patients with advanced gastrointestinal tumors is still unsatisfactory, meaning that further exploration of the prognostic value and immunoregulatory function of OTUB1 for digestive cancers is required.

Immunotherapy plays a prominent role in tumor treatment, and digestive tumors may benefit from treatments related to immunotherapy (Kather and Halama, 2019). The type and level of tumor-infiltrating lymphocytes can affect the prognosis of the tumor and the efficacy of immunotherapy (Kather and Halama, 2019; Rohaan et al., 2019). OTUB1 is an important regulator in the level and types of tumor-infiltrating immunocytes. OTUB1 regulated the anergy of T-cells via interaction with RNF128/GRAIL (Lin et al., 2009; Stempin et al., 2017) and modified the activation of CD8 T cells. As a checkpoint of IL-15-mediated priming, OTUB1 exerts a dramatic influence on the activation of CD8 T cells and NK cells (Zhou et al., 2019). The deficiency of OTUB1 could enhance the sensitization of CD8 T cells to TCR-CD28 and subsequently activate T cells into antigen-specific effector cells (Zhou et al., 2019). OTUB1 also potently regulates the function of DC and influences the synthesis of immune factors. The deletion of OTUB1 in DCs will reduce the production of protective IFN-γ secreted by NK cells (Mulas et al., 2020). OTUB1 can promote the activity of the NF-κB pathway and the synthesis of cytokine. Furthermore, OTUB1 participated in the reprogramming of metabolic progress, which is an essential mechanism for the proliferation, generation, and function of activated T cells (Pearce et al., 2013). The above knowledge helps us understand the role of OTUB1 in immune regulation and contributes to the research of immunotherapy.

OTUB1 overexpression can elevate the expression level of P53 transcriptional targets, and subsequently activate the apoptotic regulatory pathway, depending on P53. The ubiquitination of P53 was inhibited by OTUB1 non-canonically to promote its stabilization. In detail, OTUB1 could directly bind P53 and deubiquitinates it, which may further improve the stability of P53 mediated by OTUB1, and thus influencing the response to DNA damage and the damaged-regulated autophagy induced by P53. The abnormal expression of OTUB1 attenuated the above process, and its effect was that it was able to be rescued by restoring the normal construct of OTUB1 (Sun et al., 2012; Li et al., 2014).

Autophagy is the process that can degrade and recycle unnecessary or damaged cellular components (Feng et al., 2015; Klionsky, 2018). It has been reported that P53 plays a dual role in cell autophagy according to its cytoplasmic localization in cells. Specifically, in the nucleus, P53 can positively regulate autophagy through the P53-DRAM signaling pathway, while the P53 in the cytoplasm inhibits autophagy through the mTOR signaling pathway, and the inhibitory function of P53 is independent of its transcriptional activity (Feng et al., 2005; Crighton et al., 2006; Tasdemir et al., 2008; Eby et al., 2010; Kenzelmann et al., 2013). OTUB1 exerts a contradictory effect on autophagy flux via in regulation of P53 depending on the context-specific manner.

OTUB1 might play an important role in cell autophagy and apoptosis through ATG5, which is necessary for the formation of autophagic vesicles, and the abnormal expression of ATG5 will impact the initiation of autophagy. ATG5 negatively regulated the innate antiviral immune response and MHC-II antigen presentation, and participated in the maturation and apoptosis of lymphocytes (Pierdominici et al., 2012). Thus, the regulation of ATG5 by OTUB1 might indirectly regulate autophagy and immune processes.

Due to the functional complexity of OTUB1 in autophagy and immune response, researchers need to be more careful when evaluating the prognostic value of OTUB1 in different cancers. More molecular and animal studies are needed to verify the prospects for the clinical application of OTUB1 on tumor progress. Above all, our study analyzed the efficacy of OTUB1 in immunization regulation and the prognosis of gastrointestinal tumors, but further mechanism research and clinical validation are still needed. This research provides an important bioinformatics reference that will benefit future research into mechanisms and potential clinical applications. OTUB1 is a potent novel immunotherapy and antitumor target for digestive cancers.

## Data Availability Statement

The raw data supporting the conclusions of this article will be made available by the authors, without undue reservation, to any qualified researcher.

## Author Contributions

WQ designed this study and wrote the manuscript. WQ and WZ extracted the information from the databases. WZ analyzed the data. Both authors revised the manuscript.

## Conflict of Interest

The authors declare that the research was conducted in the absence of any commercial or financial relationships that could be construed as a potential conflict of interest.

## Footnotes

1 https://www.oncomine.org/resource/login.html

2 https://cistrome.shinyapps.io/timer/

3 http://kmplot.com/analysis/

4 http://gepia.cancer-pku.cn/index.html

5 https://www.pathwaycommons.org

## Abbreviations

CHOL: cholangiocarcinoma
COAD: colon adenocarcinoma
DUB: deubiquitinating enzymes
ESAD: esophageal adenocarcinoma
ESCA: esophageal carcinoma
HR: hazard ratio
LIHC: liver hepatocellular carcinoma
OS: overall survival
OTUB1: Ovarian tumor domain-containing ubiquitin aldehyde binding protein 1
OTUDs: ovarian tumor protease domain-containing DUBs
PAAD: Pancreatic adenocarcinoma
READ: rectum adenocarcinoma
PFS: progression-free survival
RFS: relapse-free survival
TAM: tumor-associated-macrophage
TILs: tumor-infiltrating lymphocytes.
